# Phagophobia: a case report

**DOI:** 10.1186/1756-0500-7-574

**Published:** 2014-08-27

**Authors:** Chathurie Suraweera, Raveen Hanwella, Varuni de Silva

**Affiliations:** National Hospital of Sri Lanka, University Psychological Medicine Unit, Colombo, Sri Lanka; Department of Psychological Medicine, Faculty of Medicine, Kynsey Road, Colombo 08, Sri Lanka

**Keywords:** Phagobhobia, Fear of swallowing, Psychological dysphagia, Choking

## Abstract

**Background:**

Phagophobia is a rare disorder and the literature is sparse. There is no specific treatment described for this life threatening condition.

**Case presentation:**

The patient is a 25-year-old Sri Lankan female with recurrent difficulty in swallowing. Following her initial episode which lasted one year, she presented to us with inability to swallow for one week. She was dependent on liquids and semisolids. The medical team confidently excluded an organic cause. She had difficulty swallowing solids with behaviours like swallowing with the aid of water and swallowing small boluses. She had difficulty eating in front of a crowd as well. She was preoccupied with misconceptions related to food and gastrointestinal disorders like gastritis. The symptom was soon becoming a maladaptive coping mechanism as it occurred when she was under stress and had difficulty solving a problem. The patient was managed with graded exposure and cognitive techniques.

**Conclusion:**

The possibility of a psychological cause for dysphagia should be borne in mind although the occurrence is rare. Although no definitive treatment methods for phagophobia are described, cognitive behavioural techniques can successfully be used in the treatment.

## Background

Phagophobia is the avoidance of swallowing foods, liquids, or pills usually based on a fear of choking [1]. It is a psychological form of dysphagia and is characterized by various significant swallowing complaints with normal physical examination and investigation findings. Although many authors have used the term “choking phobia” to describe patients with phagophobia, it is a confusing term because patients are not able to distinguish difficulties with bolus propulsion versus aspiration. Although the clinicians define choking as a symptom of aspiration, patients refer to either problem as choking. Therefore, Shapiro et al. suggested the term “phagophobia”, as more suitable for such patients [2]. Phagophobic patients show fear and avoidance of swallowing food, fluids, or pills. It is recognized in the Diagnostic and Statistical Manual of Mental Disorders (DSM-IV) as a specific phobia in the residual category (i.e. “other”) along with phobias of vomiting or contracting an illness [3]. Effects of phagophobia can include weight loss and avoidance of eating resulting in malnutrition. It is a rare and disabling condition of non-organic aetiology which at times can be severe enough to be life threatening. We describe a case of successful management of phagophobia with cognitive behavioural techniques.

## Case presentation

The patient is a 25-year-old Sri Lankan female who developed fear of swallowing. This was following an incident where she choked a mouthful of rice during an episode of gastritis. She found the incident painful due to acidic regurgitation that followed. The initial episode lasted for over one year during which she was totally dependent on fluids and semisolids which lead to significant weight loss. During this period, she was extensively investigated for dysphagia which included several invasive methods. She recovered temporarily but with waxing and waning of symptoms. She was on several psychotropic medications including antipsychotics.

At the time of presentation, she had not eaten for one week and was only on liquid and semisolid diet. She had intensive craving for food and inability to swallow solid food and pills. She avoided food and had intrusive images of the first choking episode whenever she brought food close to her mouth. She also had anticipatory anxiety prior to meal times.

The patient had several behaviours like, consuming fluids and semisolids, monitoring every step of swallowing, monitoring anxiety symptoms like palpitations and tachypnea, eating solids in small mouthfuls, swallowing one mouthful in several attempts, frequent drinking of water to facilitate swallowing and avoiding eating in social situations.

Additionally it was identified that the patient had misconceptions about swallowing, food and gastritis. For example she thought that foods she did not like to eat caused gastritis and that food should be eaten warm.

Her condition worsened with stress and the symptoms recurred whenever she experienced strong emotions like anger or sadness. The high expressed emotion of family members and the stigma of being thin as she had lost a considerable amount of weight contributed to her anxiety and maintained the symptoms. She did not have features suggestive of panic disorder, generalized anxiety disorder or psychosis.

The management of her condition was multifaceted. First she was educated about the mechanism of swallowing, food with high calorie and low calorie intake and gastritis. The subsequent steps in therapy were relaxation training particularly of the head and neck muscles, strengthening of the coughing mechanism in case of an actual event of choking, distraction from self-monitoring of stages of swallowing and replacing the image of the initial choking episode with a pleasurable image.

She was gradually exposed to different eating situations. The situations in order were eating with the therapist, family members, friends, social gatherings and strangers. She was also exposed to food that she was preoccupied with and to swallowing of pills. Graded exposure was done for the amount of food eaten, gradually increasing the size of mouthfuls and reducing the number of times she swallowed each mouthful. Cognitive therapy was done to restructure her cognitive errors and maladaptive thinking along with coping skills training.

The patient improved steadily with the above methods. She gradually regained the ability to swallow small food boluses aided by water by the end of first week. She was seen daily during this period. She was later able to swallow much larger boluses in front of others as well. With improved confidence in swallowing, cognitive therapy for the correction of faulty assumptions, and education of family members to carry out therapy, her anxiety subsided. The stress management techniques and better coping strategies helped prevent relapse at stressful times. Her session frequency was decreased to twice weekly by the second week and later to weekly. She recovered in three months which consisted of 20 sessions of therapy. Currently, she has been asymptomatic for more than one year and did not relapse even after several stressful life events.

## Conclusion

It is important to differentiate phagophobia as a psychogenic dysphagia from organic dysphagia. The patient we describe was repeatedly investigated and an organic cause excluded by a physician prior to referral. Our patient complained of food being stuck at the entrance to the pharynx and also it not being propelled beyond. However, on further inquiry she revealed that it is the ‘feeling’ of food getting stuck. She did not require maneuvers to dislodge the bolus. As the barium swallow was also normal, the condition was diagnosed as phagophobia. Organic (non-psychogenic) dysphagia is secondary to problems with either bolus propulsion or aspiration. In the case of abnormal bolus propulsion, patients will feel the food getting stuck. The passage of the bolus will take place spontaneously after some time or will have to be aided by liquids, multiple swallowing attempts or even pharyngeal regurgitation as in our patient [4].

Phagophobia also requires differentiation from psychiatric disorders which include swallowing dysfunction, such as conversion disorder, anorexia nervosa and bulimia nervosa. Globus is distinguished from phagophobia by the ‘perception’ patient has of noninterference with actual swallowing. Some patients with conversion disorders may present as globus; however, this is usually unrelated to actual swallowing. Conversion disorder was ruled out because our patient did not get globus pharyngeus. The sensation of her throat tightening and the feeling that food became lodged in the throat were experienced only during meal time. Patients with phagophobia lose weight due to avoidance of food. However, it is different from eating disorders as they do not avoid eating to lose weight but are keen on gaining the weight they have lost.

McNally proposed that phagophobia is most often secondary to conditioning experience like choking on food as in the case of our patient [5]. It is at times precipitated by witnessing a choking incident. Although no controlled trials have evaluated treatments for phagophobia, there are case studies describing a diversity of treatment approaches. Although pharmacotherapy has been used in the treatment of phagopobia, behavioral approaches are more common. For example, Ball and Otto [6] used a treatment protocol consisting of psycho-education, cognitive restructuring, aversion/distraction and in vivo exposure with three adult patients and reported positive results in all three patients following 11 to 13 sessions.

The management of our patients was based on cognitive behavioural techniques. She improved after three weeks of intensive therapy with daily supervision in the initiating week and remained well after one year.

## Consent

Written informed consent was obtained from the patient for publication of this case report. A copy of the written consent is available for review by the Editor-in-Chief of this journal.
